# The efficacy and cerebral mechanism of intradermal acupuncture for major depressive disorder: a study protocol for a randomized controlled trial

**DOI:** 10.3389/fpsyt.2023.1181947

**Published:** 2023-05-15

**Authors:** Xiaoting Wu, Mingqi Tu, Nisang Chen, Jiajia Yang, Junyan Jin, Siying Qu, Sangsang Xiong, Zhijian Cao, Maosheng Xu, Shuangyi Pei, Hantong Hu, Yinyan Ge, Jianqiao Fang, Xiaomei Shao

**Affiliations:** ^1^Key Laboratory for Research of Acupuncture Treatment and Transformation of Emotional Diseases, The Third Clinical Medical College, Zhejiang Chinese Medical University,, Hangzhou, China; ^2^The Third Affiliated Hospital of Zhejiang Chinese Medical University, Hangzhou, China; ^3^The First Affiliated Hospital of Zhejiang Chinese Medical University, Hangzhou, China

**Keywords:** major depressive disorder, intradermal acupuncture, cerebral mechanism, magnetic resonance imaging, magnetic resonance spectroscopy, selective serotonin reuptake inhibitors

## Abstract

**Background:**

Major depressive disorder (MDD) has emerged as the fifth leading cause of years lived with disability, with a high prevalent, affecting nearly 4% of the global population. While available evidence suggests that intradermal acupuncture may enhance the effectiveness of antidepressants, whether its efficacy is a specific therapeutic effect or a placebo effect has not been reported. Moreover, the cerebral mechanism of intradermal acupuncture as a superficial acupuncture (usually subcutaneous needling to a depth of 1–2 mm) for MDD remains unclear.

**Methods:**

A total of 120 participants with MDD will be enrolled and randomized to the waiting list group, sham intradermal acupuncture group and active intradermal acupuncture group. All 3 groups will receive a 6-week intervention and a 4-week follow-up. The primary outcome will be measured by the Hamilton Depression Rating Scale-17 and the secondary outcome measures will be the Self-Rating depression scale and Pittsburgh sleep quality index. Assessments will be conducted at baseline, 3 weeks, 6 weeks, and during the follow-up period. In addition, 20 eligible participants in each group will be randomly selected to undergo head magnetic resonance imaging before and after the intervention to explore the effects of intradermal acupuncture on brain activity in MDD patients.

**Discussion:**

If the intradermal acupuncture is beneficial, it is promising to be included in the routine treatment of MDD.

**Clinical Trial Registration:**

Clinicaltrials.gov, NCT05720637.

## Background

Major depressive disorder (MDD) is a mental disorder characterized by behavioral, cognitive, and emotional changes. It has emerged as the fifth leading cause of years lived with disability (GBD 2016 Disease and Injury Incidence and Prevalence Collaborators, 2017), affecting nearly 4% of the global population with an annual incidence of approximately 290 million cases (1). Symptoms of MDD include low self-esteem, loss of interest or pleasure, social withdrawal, poor concentration, insomnia, poor appetite or overeating, and in severe cases, self-harm and suicide (2, 3). Given its chronicity and high relapse rate, MDD is difficult to treat and has a serious long-term impact on the quality of life of patients. It has been regarded as a major public health concern.

Given the complexity of genetic and environmental factors, several hypotheses for the pathogenesis of MDD have been derived. Available evidence suggests that genetic mutations, oxidative stress, chronic inflammation, peripheral hormone-type factors dysregulation, and neurotransmitter dysfunction (e.g., serotonin 5-HT) may drive the development of MDD (4–6). For example, in a chronic mild stress model of MDD, structural damage in the hippocampus, hypothalamus and cortex could be observed, which may have contributed to the depression-like behavior (7–9). The prefrontal cortex (PFC) plays a critical role in the interaction between the central and the autonomic nervous system (10, 11). Dysfunction of the PFC results in impairment of emotional memory circuits and aversive learning, followed by chemical dysregulation, ultimately leading to cognitive changes and disturbed behavioral responses in MDD (12, 13). What is more, the monoamine hypothesis is widely regarded as the prevailing theory in the pathogenesis of MDD, with particular emphasis on the 5-HT hypothesis (14, 15). This point that reduced 5-HT levels increase the risk of developing MDD, which provides the underlying rationale for selective serotonin reuptake inhibitor (SSRI) as the frontline antidepressant (4, 16). Nevertheless, a recent systematic review has cast doubt on the consistency of evidence suggesting that MDD is linked to or caused by decreased 5-HT activity (17). Notably, long-term antidepressant administration may even lead to a decrease in plasma 5-HT, adding to the confusion surrounding the actual relationship between MDD and 5-HT (18–20).

When it comes to the treatment of MDD, it is based on symptom control and devoted to restoring the patient’s psychological and physical function to baseline levels (21). Clinical evidence-based treatments for MDD consist of antidepressants such as selective serotonin reuptake inhibitors (SSRIs), as well as psychological interventions such as counseling, behavioral activation, cognitive behavioral therapy, and interpersonal therapy (22, 23). Generally, SSRIs are the frontline antidepressant for MDD with overall effectiveness, but they have some limitations, including delayed onset, inadequate response, side effects, drug resistance, or withdrawal syndromes in long-term use, which negatively impact treatment outcomes (24, 25). Or worse, instead of achieving remission, some patients exacerbate emotional or somatic symptoms, increasing the risk of comorbidities (26, 27). While newer antidepressants, such as Vilazodone and Vortioxetine, may improve the tolerability and efficacy with a lower risk of adverse effects, they are often prohibitively expensive and not available in generic formulations, making it challenging to meet the daily needs of MDD patients (2, 28). Hence, given the limitations of current antidepressants, there is a pressing need for new (combination) treatments that can improve efficacy and safety.

One such treatment option is acupuncture, a component of Traditional Chinese Medicine that has been used internationally for the treatment of MDD (29, 30). Treatment with acupuncture alone or in combination with appropriate adjuncts has been reported to be significantly effective in reducing the severity of MDD, relieving patients’ somatic symptoms, and improving sleep (31, 32). Compared to SSRIs-only, acupuncture combined with SSRIs for MDD has a faster onset of effect and fewer side effects (33). Despite these promising findings, the quality of these studies was poor. As noted by the Cochrane review, there was no evidence of differences in adverse effects between acupuncture and control acupuncture/usual treatment in most studies (29). Moreover, effect expectations and placebo effects have not been clearly elucidated, and these might lead to an overestimation of the actual therapeutic effects of acupuncture. In this way, it is essential to evaluate the effect expectations, integrity of blinding, incidence of adverse events rate, and treatment adherence in both the acupuncture and controls to improve the quality of the study.

Among the various acupuncture methods, intradermal acupuncture (IA) is a treatment method that uses short indwelling needles retained under the skin to produce continuous stimulation for long-lasting efficacy (34). This method is characterized by its ease of operation, painlessness, convenience, and negligible interference with the patient’s daily activities. It can facilitate patient compliance and treatment effectiveness, making it an ideal choice for treating chronic diseases such as insomnia and MDD (34, 35). Evidence from studies supported that IA can significantly reduce the Pittsburgh sleep quality index (PSQI) scale scores and effectively improve insomnia symptoms (35, 36). Insomnia is one of the key risk factors for MDD, and while IA has been studied for its efficacy in alleviating insomnia, there is limited research on its therapeutic effects and mechanisms on MDD (37, 38). One preliminary clinical study revealed that IA could improve Beck Depression Inventory scores in patients with MDD (37), and another demonstrated that at 2 weeks after the intervention, patients in the AIA combined with SSRIs group showed a significant decrease in the Hamilton Depression Rating Scale-17 (HAMD-17) and the Self-Rating Depression Scale (SDS) scores compared to the SIA combined with SSRIs group, indicating that IA enhanced the antidepressant efficacy of SSRIs (38). However, both studies had limitations such as small sample sizes, short observation periods, and no investigation of medication side effects. Thus, high-quality clinical studies on the efficacy of IA for MDD are still lacking.

Functional magnetic resonance imaging (fMRI) is a common neuroimaging method with characteristics of non-invasive, non-radiation exposure and high spatial resolution for observing brain activity in human (39). There are 3 methods to analyze fMRI data to present the homogeneity and spontaneous activity of the brain: functional connectivity (FC) describes the synchronicity between different regions, while regional homogeneity (ReHo) and amplitude of low frequency fluctuation (ALFF) describe the homogeneity and intensity of regional activity, respectively (40). In MDD, emotion-related brain regions mainly include the PFC, anterior cingulate cortex (ACC), amygdala, hippocampus, and hypothalamus. Compared to healthy controls, MDD patients showed abnormalities in these regions in ALFF, ReHo, and FC, which may be related to the development and severity of MDD (41–44). These findings suggest that impaired function of specific brain regions may be associated with the development and severity of MDD. In addition, abnormal levels of γ-aminobutyric acid (GABA) and glutamate acid (Glu) have been found in the ventral PFC/ACC of MDD patients based on magnetic resonance spectroscopy (MRS), an fMRI-based technique commonly used to detect levels of multiple neurotransmitters or metabolites in the brain (45, 46).

However, there were few studies on the therapeutic mechanisms of IA and no neuroimaging evidence. As previously mentioned, fMRI has been used not only to observe activity in specific brain regions in MDD patients, but also to quantify the brain response induced by acupuncture for MDD in an attempt to illuminate the cerebral mechanism (47). Importantly, the activity in brain regions such as the ACC, hypothalamus, amygdala and dorsolateral PFC, was found to be positively correlated with the efficacy of acupuncture for MDD, suggesting that acupuncture can improve depressive symptoms by modulating brain networks (47–49). What this evidence focused on, however, was the cerebral mechanism of manual and electroacupuncture for MDD, and not IA. To date, there has been no report on whether the cerebral mechanisms of IA as superficial acupuncture (usually needling to a depth of 1–2 mm subcutaneously) for MDD are consistent with those of deep acupuncture. Previous studies have revealed that the effects of acupuncture are strongly influenced by the depth of needling, as the cutaneous and deep tissues innervated by the somatosensory system show different densities of innervation (50, 51). Additionally, Feng et al. analyzed the brain network connectivity in patients with mild cognitive impairment who received deep or superficial stimulation using fMRI, and found significant differences in correlations between specific brain regions (52). These studies have provided insight into the differing mechanisms of treatment with deep and superficial acupuncture. As such, the antidepressant mechanism of IA needs to be further explored.

Together, we design a randomized controlled trial (RCT) with two aims. First, the clinical efficacy and safety of AIA for MDD will be evaluated by comparing with the SIA/waiting list. Second, the effects of AIA on brain activity of MDD patients will be detected by fMRI and MRS, and the correlation between the effects and clinical variables will be analyzed to initially explore the cerebral mechanisms of AIA on MDD.

## Methods

This study is a 10-week trial with a randomized block method in which patients with MDD will be allocated in a balanced ratio (1:1:1). This study protocol is reported based on the Standard Protocol Items: Recommendations for Intervention Trials (SPIRIT) statement. More, this study has been approved by the Medical Ethics Committee of the Third Affiliated Hospital of Zhejiang Chinese Medical University (approval number: ZSLL-KY-2022-001–01-01), and is registered within the ClinicalTrials.gov Identifier: NCT05720637.

### Study design

**Figure 1:**
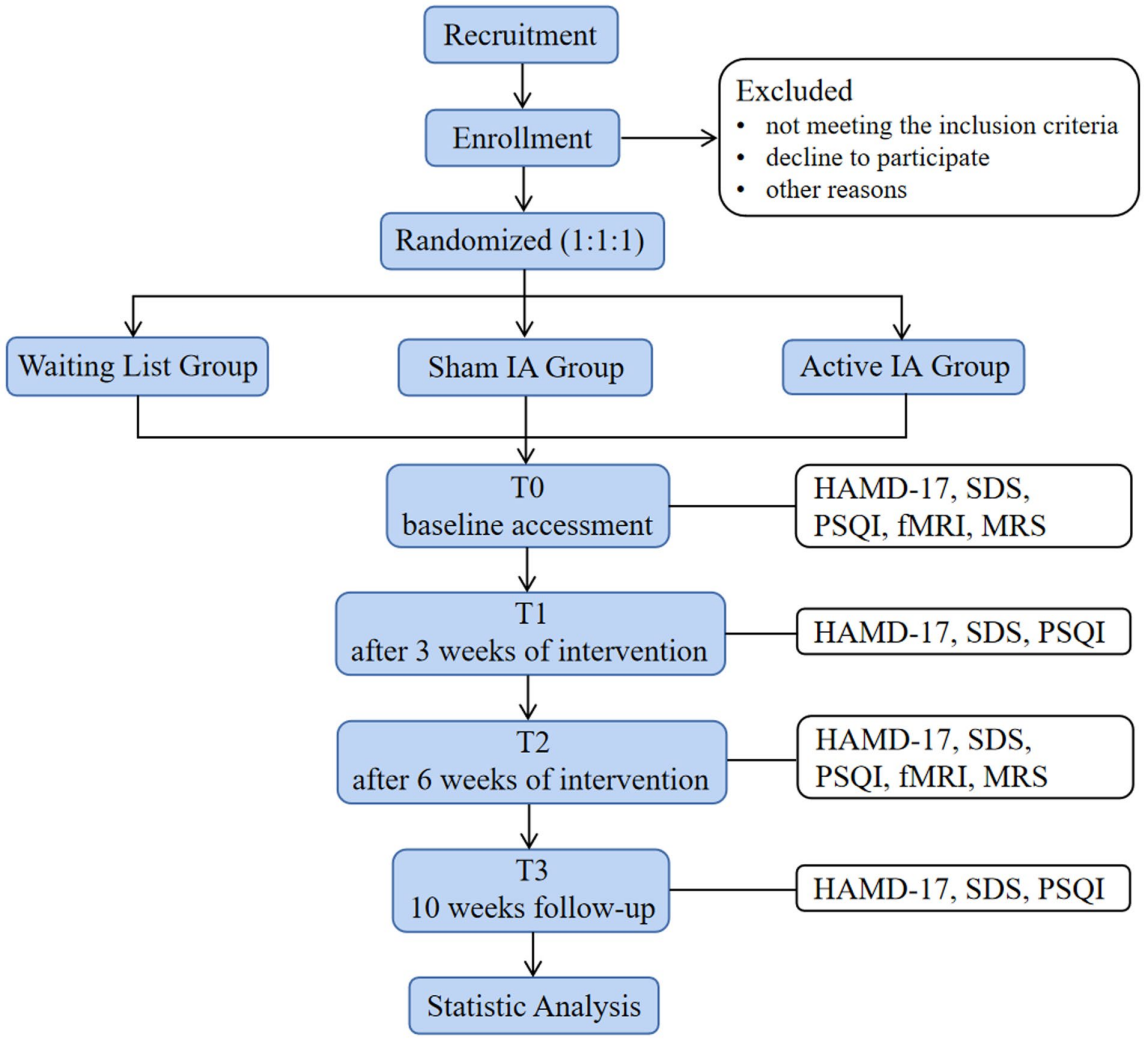
Flowchart of this study.

This study is a double-blind, randomized controlled trial. Participants will be randomized into waiting list group (patients in this group will be treated with SSRIs only), sham intradermal acupuncture combined with SSRIs (SIA) group and active intradermal acupuncture combined with SSRIs (AIA) group in a 1:1:1 allocation ratio. The flow chart shown in Figure 1 illustrates the trial in more detail.

### Setting

The trial will be conducted at the Third Affiliated Hospital of Zhejiang Chinese Medical University, Tongde Hospital of Zhejiang Province and Hangzhou First People’s Hospital.

### Participant recruitment

A total of 120 eligible participants will be recruited from three centers. The recruitment will be advertised online or offline and participants can contact the researcher via WeChat or telephone. Eligible volunteers will be invited to participate in the study. After confirming the details of the study process, they will sign an informed consent form and undergo a baseline assessment.

### Sample size

Combining the relevant literature with our preliminary pre-experiment, it was predicted that the mean reduction in HAMD for each group at the end of the intervention would be 12.1, 9.7, and 9.5 with standard deviations of 2.7, 3.7, and 3.2, respectively. When α = 0.05, 1-β = 0.9 and the sample sizes of the 3 groups are equal, a sample size of 32 per group is calculated using the PASS 15 software. Allowing for the possibility of loss and attrition (20%), 40 subjects per group, for a total of 120, is reasonable.

In addition, relatively stable results could be obtained in neuroimaging studies with 20 subjects per group (53, 54). Therefore, 20 eligible subjects from each group will be randomly selected and undergo head MRI scans before and after the intervention.

### Eligibility criteria

#### Inclusion criteria

Participants with all of the following criteria will be included:

(1) Patients diagnosed with MDD according to the International Classification of Disease-10 (ICD-10); HAMD-17 ≥ 17;
(2) Aged between 18 and 60 years (no limitation on gender);
(3) Administration of SSRIs at least 6 weeks;
(4) Patients undergoing MRI and MRS should be right-handed and free of traumatic brain injury, claustrophobia or metal implants;
(5) Written informed consent is obtained by the person.

#### Exclusion criteria

Participants with any of the following criteria will be excluded:

(1) ICD-10 diagnoses: schizophrenia, bipolar disorder, manic episode or other psychotic disorders; alcohol and drug addiction; a current substance use disorder and lifetime history of substance abuse;
(2) Significant skin lesions, severe allergic diseases, tumors, and severe or unstable internal diseases involving the cardiovascular, digestive, endocrine, or hematological system;
(3) Positive suicidal tendency (suicidal intent and recent suicidal behavior) is determined by any affirmative response to items 5 or 6b or 7b of the Columbia-suicide severity rating scale;
(4) Allergy to adhesive tape, fear of intradermal acupuncture;
(5) Pregnancy and lactation;
(6) Mental retardation and difficulty cooperating with doctors;
(7) Previously treated with intradermal acupuncture or participating in other clinical trials.

### Withdrawal criteria and management

#### Withdrawal or dropout criteria

(1)Serious adverse reactions or other unexpected events that make continued participation in the study inappropriate.
(2)Difficulty in cooperation and poor compliance.
(3)Participants use a treatment that is prohibited in this study or does not follow medical advice to change the dose of medication.
(4)Participants have a serious adverse reaction related to acupuncture treatment.
(5)Participants voluntarily withdraw from the study, but those receiving more than 1/2 session should be counted in the efficacy statistics.

#### Withdrawal management

Details of all withdrawn participants should be recorded on the case report files (CRF) to ensure credibility and transparency. For participants who withdraw due to adverse reactions or unexpected events, the researcher should make a detailed record on the CRF and the psychiatrist should take appropriate measures according to the participant’s actual condition. Once participants are enrolled in the trial in order, those receiving more than 1/2 sessions should be counted in the efficacy statistics. An intentionality analysis will be conducted on all eliminated and withdrawn participants.

### Randomization and allocation concealment

Eligible participants will be assigned to the waiting list group, SIA group and AIA group with a random block scheme using SPSS 25 (SPSS Inc., Chicago, IL, United States) software, which will be stratified by the sex and age. The block size will adopt 6. Then, the random numbers will be placed in sealed envelopes by an independent assistant, and provided to the participants after the baseline assessment.

### Blinding

Participants, outcome assessors and statisticians will be blinded to the group allocation. Due to the proprietary treatment modalities of acupuncture, it is not possible to blind acupuncturists, and therefore they will be instructed not to communicate with participants about the treatment allocation. Acupuncture will be performed in an isolatable room to avoid communication between participants, thus ensuring a good implementation of the blinding method.

### Intervention

Medication administration and acupuncture will be performed by certified psychiatrists and acupuncturists, respectively. Acupuncturists will receive specific training to fully understand the standardized operation, including acupoints selection and positioning, acupuncture manipulation and frequency.

### Antidepressant medication

All eligible participants will be treated with oral SSRIs. Before being included, participants should have been taking SSRIs for at least 6 weeks to achieve stable antidepressant treatment, and those who do not respond well to SSRIs will be excluded. For participants not taking SSRIs before this trial, the medication dose will be increased to the recommended level (20 mg/d for fluoxetine, paroxetine and citalopram, 10 mg/d for escitalopram, 50 mg/d for sertraline and 100 mg/d for fluvoxamine) (55). This dosing regimen has been widely used in China. If the participant tolerates the initial SSRI medication poorly, the psychiatrist will switch the prescription to another SSRI according to the medication protocol, and they should be in an antidepressant regimen stable before enrollment. Moreover, the researcher should not change the antidepressant regimen during the trial. Although temporary administration of Valium is allowed, it should be approved by a psychiatrist and recorded in detail on the CRFs. Participants will be instructed to keep a medication diary for the psychiatrist to assess compliance with treatment.

### Waiting list

Participants randomly assigned to the waiting list group will be treated with SSRIs only for 6 weeks, they could choose 6 weeks’ intradermal acupuncture treatments free of charge after the clinical trial.

### Active intradermal acupuncture

**Figure 2:**
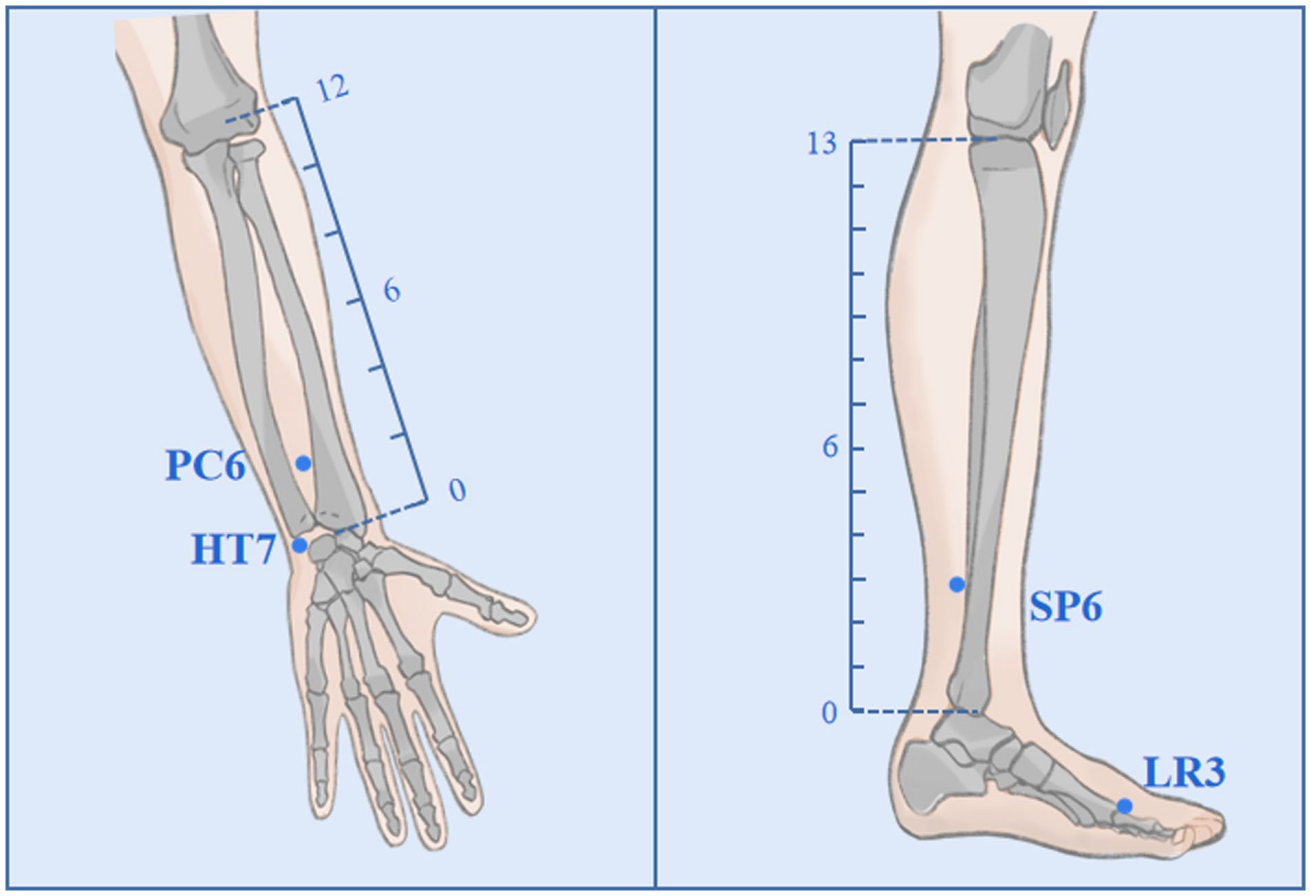
Acupoints and locations. The locations of Neiguan (PC6), Shenmen (HT7), Sanyinjiao (SP6), and Taichong (LR3).

Participants randomly assigned to the AIA group will receive an acupuncture intervention for approximately 6 weeks, once every 4 days, for a total of 10 times. In Traditional Chinese Medicine (TCM) theory, the principles of treatment for MDD are dispersing stagnated hepatoqi and tranquilize the mind. Therefore, 4 commonly used acupoints (all taken bilaterally) with such effects will be selected as targets in this trial, namely Shenmen (HT7), Neiguan (PC6), Sanyinjiao (SP6) and Taichong (LR3); (Figure 2). Depending on the location of the acupoint, a φ0.20*1.5 mm or φ0.20*1.2 mm AIA (SEIRIN Co., Japan) will puncture perpendicularly and retained in the skin. It will be retained for 72 h and removed and rested for 1 day. During the retention period, participants will be asked to apply pressure 3–4 times a day for approximately 1 min at 4-h intervals, stimulating as much as tolerated (Table 1).

**Table 1:** Details on active IA and sham IA.

No. Acupoint		Active intradermal acupuncture			Sham intradermal acupuncture		
		Method	Frequency	Needle sizes	Method	Frequency	Needle sizes
1	Taichong (LR3)	Punctured perpendicularly and retained in the skin, applying pressure 4 times a day.	Retained for 72 h each time,10 times in total.	φ0.20*1.5 mm	Attached to the skin surfacewithout applying pressure.	Retained for 72 h each time,10 times in total.	—
2	Neiguan (PC6)			φ0.20*1.2 mm			
3	Sanyinjiao (SP6)			φ0.20*1.5 mm			
4	Shenmen (HT7)			φ0.20*1.2 mm			

### Sham intradermal acupuncture

**Figure 3:**
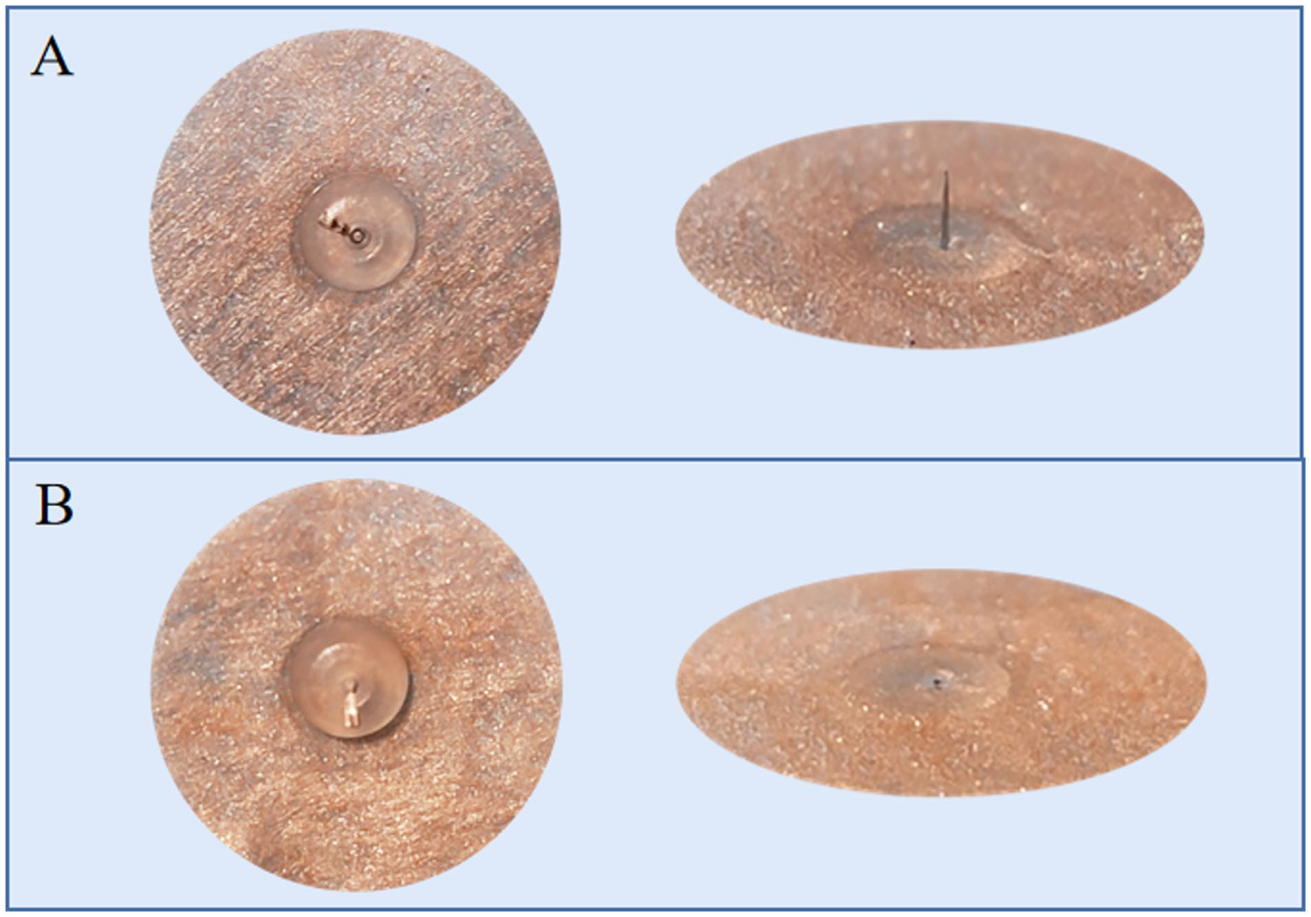
Active intradermal acupuncture (AIA) and sham intradermal acupuncture (SIA). **(A)** shows the AIA with an intradermal needle, while **(B)** shows the SIA with a thin silicone pad.

The SIA (SEIRIN Co., Japan) has the same size, color and material as the AIA, but with a thin silicone pad in the middle instead of a needle body (Figure 3). SIA will also be attached to the acupoints and retained for 72 h, then removed for a day’s rest (Table 1). A total of 10 treatments will be administered over 6 weeks.

### Outcomes

Table 2 shows details of assessments at enrollment, allocation, treatment, and follow-up periods.

**Table 2:** Assessment during enrollment, allocation, treatment, and follow-up.

	Screening and baseline	Treatment		Follow-up
Timepoint	Week 0	Week 3	Week 6	Week 10
Eligibility Criteria	**×**			
Consent	**×**			
Allocation	**×**			
Interventions:				
Active IA	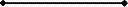			
Sham IA	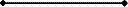			
Waiting list	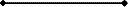			
Assessments:				
HAMD-17	**×**	**×**	**×**	**×**
SDS	**×**	**×**	**×**	**×**
PSQI	**×**	**×**	**×**	**×**
MRI	**×**		**×**	
MRS	**×**		**×**	
Acupuncture expectation	**×**			
Acupuncture compliance			**×**	
Blind success rate evaluation			**×**	
Adverse events		**×**	**×**	**×**
Medication record		**×**	**×**	**×**

#### Primary outcome

Changes in the Hamilton Depression Rating Scale-17 (HAMD-17) Scores.

In this trial, changes in HAMD-17 scores measured between baseline and 6 weeks of treatment will be defined as the primary outcome. The HAMD-17 is used to assess the severity of clinical depressive symptoms and the effectiveness of treatment before and after clinical trials (56, 57). It includes anxiety/somatization, cognitive impairment, retardation, sleep disorder and weight changes. A higher HAMD-17 score indicates a greater severity of MDD symptoms, which are classified into the following four levels: 0–7 is normal, 8–16 is mild, 17–23 is moderate and 24 or more is severe (58).

#### Secondary outcome

Secondary outcomes are MDD symptoms, sleep quality and adverse events during the intervention. They include the changes in SDS (week 3, 6 and 10) and PSQI (week 3, 6 and 10) compared to the baseline period. Also, changes from baseline in HAMD-17 scores at weeks 3 and 10 will be measured.

The SDS items are chosen based on depression symptom factor analytic investigations (59). The standard score is the total of all scores multiplied by 1.25 to the nearest whole number on this scale, which has 20 questions. The SDS is designed in such a way that the less depressed patients’ chief complaint scores are lower, while the more depressed patients’ chief complaint scores are higher. A standard score below 50 is normal; 50–59 is mild; 60–69 is moderate; 70 or more is severe (60). The SDS is frequently used in the assessment of clinical MDD due to its high sensitivity and good internal consistency (61).

The PSQI is a self-assessment questionnaire developed by psychiatrist Dr. Buysse in 1989 and is commonly used to assess the quality of sleep during the preceding month (62). It has 19 items covering 7 factors, namely subjective sleep quality, latency, duration, efficiency, disorders, medication use, and daytime dysfunction, with a total score of 21. Higher scores on the PSQI indicate poorer quality of sleep, with a threshold score of 7.

### MRI and MRS data acquisition

For getting high-quality MRI scans, not only will the radiologist receive standardized training, but the participants will also be given uniform instructions. Specifically, during the MRI and MRS test, patients will be instructed to lie flat and relaxed, as well as the radiologist will stabilize the patient’s head with a head mask and sponge, and put earplugs in their ears to reduce noise stimulation from the device. Patients are scanned before and after the treatment session (week 0 and week 6, respectively).

All scans will be performed with a 3.0 Tesla MR scanner (GE Discovery MR750, GE Healthcare, Chicago, IL, United States). The 8-channel head coil is used for signal reception to obtain the T1-weighted structural image and echo-planar T2^*^-weighted image (EPI). MRI and MRS data acquisition will be performed in the absence of definite intracranial abnormalities after routine serial axial scans have ruled out intracerebral organic lesions or pseudo-images.

Structural images will be acquired by 3D T1BRAVO sequence: time of repetition (TR) = 8.2 ms, time of echo (TE) = 3.2 ms, flip angle = 12, field of view (FOV) = 256 mm × 256 mm, matrix = 512 × 512, and slice thickness = 1 mm. Resting state functional data will be obtained via EPI for 240 time points in the sequence: TR = 2000 ms, TE = 35 ms, flip angle = 90, slice thickness = 5 mm, slice gap = 1 mm, FOV = 256 mm × 256 mm, and matrix = 64 × 64. _1_H-MRS will be performed using point-resolved echo spin spectroscopy (PRESS) with single voxel multiple acquisitions, the region of interest (ROI) is proposed to be the hypothalamus, and scan parameters of TR = 2,200 ms, TE = 35 ms, voxel size = 13 mm × 12 mm × 10 mm, total number of scans = 64, number of excitations (NEX) = 8, automatic machine homogenization, water suppression. Pre-scan auto-leveling will require full width half height (FWHM) <13 Hz and water suppression >95%. The functional images will be pre-processed using the software DPARSF, and the metabolite spectra will be processed and analyzed through the LCModel.

In addition, with the increased accessibility of brain MRI techniques in scientific research, incidental findings such as anatomic variants, white matter changes, intracranial tumors or cystic lesions have become common (63). The percentage of reported incidental findings ranged from 2 to 32%, and it is closely related to the field strength, sequence and the subject cohort (64, 65). This trial is the first study on the cerebral mechanisms of IA for MDD based on fMRI and MRS. Before and after the intervention, changes in brain activity will be analyzed to initially explore the effects of IA on the brain regions of MDD patients. Unlike clinical practice, the number of structural sequences in mechanistic studies is limited. This makes it hard to change the protocol when incidental findings are encountered during the study, therefore incidental findings will be managed by classification (64, 66). For normal variations (Category I) without clear diagnostic consequences, no reports will be made. For additional medical clarification (Category II) and emergency clarification (Category III), additional MR sequences or other methods will be performed by radiologists to confirm these incidental findings, and inform the participant of the results (64, 66). At the same time, these data that affect the analysis will be excluded.

### Evaluation of acupuncture expectation and compliance

A six-point method (0–5 point) will be used to assess acupuncture expectations before the first acupuncture intervention. The higher the score, the higher expectation of efficacy, and those who cannot believe acupuncture at all will write down why. At the end of the acupuncture intervention (end of week 6), compliance will be evaluated with the formula: Compliance Rate = (actual treatment times/total treatment times) *100%.

### Blind success rate evaluation

At the end of the last treatment, the percentage of patients in the SIA and AIA groups who consider themselves to have been treated with acupuncture will be compared.

### Adverse events

The details of all adverse events in this study will be recorded on CRFs, and the patients will be properly and timely managed. Adverse events such as bleeding, hematoma, or unbearable pain caused by the needle, as well as drug-induced nausea, vomiting or dizziness, will all be recorded. Serious adverse events will be reported to the ethics committee.

### Quality control and data management

Before the conduct of the formal trial, professional clinical training will be provided to familiarize researchers with the process and focus on implementation details. They will sign a standard operating procedures document and relevant confidentiality commitment. The quality of the data for all enrolled participants will be checked regularly. The researcher will submit CRFs to the data management center, where the clinical research associate (CRA) will check for accuracy and issue queries on inconsistent data, which will then need to be clarified by the researcher.

To ensure the authenticity, completeness and accuracy of the trial data, researchers will be instructed to record participants’ data on the original paper CRFs following the protocol.

### Statistical analyses

All analyses will be performed by blinded statisticians using the IBM SPSS 25.0 software. MRI data are pre-processed using DPARSF software for functional connectivity images, while MRS data are pre-calibrated using LCModel software. Normally distributed continuous variables will be presented as the mean ± SD, and non-normal variables as the median and interquartile range. Generally, the Kruskal-Wallis test will be used to compare the inter-group changes among all three groups, the Chi-square (*χ*^2^) test will be used for the categorical variables, and the generalized (logit) mixed modeling for the repeatedly measured data. Statistical significance will be defined as a value of *p*<0.05.

## Discussion

MDD is characterized by a high incidence, low cure rate and high recurrence rate. It lacks effective and safe treatment options. Although numerous studies have shown acupuncture is effective in attenuating antidepressant side effects and alleviating depressive symptoms, its treatment specificity is often questioned owing to poor trial design and management, making it difficult to provide robust conclusions. Consequently, there is an urgent need for a high-quality RCT to be conducted.

The aim of this study is to evaluate the efficacy and safety of IA for MDD. To minimize potential sources of bias and imprecision that could affect clinical outcomes, several measures will be implemented. Firstly, participants will be recruited from multiple centers and randomly assigned to the AIA, SIA, and waiting groups in a 1:1:1 ratio, thus reducing selection bias. Secondly, to ensure blinding and reduce performance bias, sham intradermal needles that are indistinguishable in appearance from the active will be used, and only participants who have not previously received IA will be included in the study. Additionally, participants in the acupuncture group (both SIA and AIA groups) will be surveyed to confirm successful blinding at the end of the intervention. While acupuncturists cannot be blinded, they will be instructed not to reveal any information about group assignments to participants. Thirdly, another potential source of bias is the effects of patient expectations and compliances. Therefore, these factors will be quantified in the acupuncture group at the start and end of the intervention to mitigate their impact on the true therapeutic effects of IA. Finally, standardized outcome assessments will be used to reduce the risk of imprecision. For instance, both clinician-administered (HAMD-17) and self-reported (SDS) scales will be utilized to assess the improvement of MDD symptoms, reducing the potential for measurement error on a single scale. Outcome assessors will receive uniform training prior to the trial and will be blinded to group assignment, which may minimize inter-assessor variability. Overall, the measures described above in this study design will help reduce the risk of bias and imprecision, resulting in a more robust assessment of the efficacy of IA for MDD. However, it is worth noting that no study can be entirely free of bias, and the results of this study will be interpreted with caution and validated by future studies.

Furthermore, due to the portability and simplicity of IA, it is almost independent of the operating environment and can be widely applicable to MDD. IA can be self-administered at home under the guidance of acupuncturists, which will greatly promote patient compliance and may be a promising treatment option for the management of MDD. Indeed, availability and generalizability of IA may be limited by factors such as age differences, cultural differences, access to healthcare, and trained practitioners, which require further investigation.

## Conclusion

To conclude, in this trial, our focus will be on comparing the therapeutic differences among the AIA group, SIA group and wait list group to investigate whether AIA positively affects MDD, as well as the enhanced effects and safety when combined with SSRIs, ultimately clarifies whether the effect of IA is specific or placebo. Furthermore, we will explore the effects of IA on emotion-related brain networks based on MRI in an attempt to explain the therapeutic mechanisms of IA. By this way, this study will provide high-quality clinical evidence for IA for MDD, identify the actual efficacy of IA and promote its clinical application.

### Limitations and future directions

This trial has the following limitations. First, patients will be recruited in only one region, Hangzhou (the provincial capital city of China), which may introduce regional bias as an influencing factor. Second, the sample size is relatively small and a larger sample size will be needed to improve the study’s power. Third, a detailed subgroup analysis of MDD patients is not performed, and the degree of MDD as well as role of comorbid personality disorders will be studied as influencing factors in the future. Fourth, the exploration of the cerebral mechanisms of IA for MDD in this study is only preliminary. Due to the limitation of scanning time, the region of interest for performing MRS will be hypothalamus only. It will be extended to more emotion-related regions in future studies. Fifth, given the characteristics of MDD and to avoid patient questionnaire fatigue, only the HAMD-17, SDS, and PSQI scales will be used. Future research will employ additional measures, such as the Back Depression Inventory and Quality-of-Life assessments, to assess MDD symptoms.

## Ethics statement

The studies involving human participants were reviewed and approved by Ethics Committee of the Third Affiliated Hospital of Zhejiang University of Traditional Chinese Medicine, the main center (ZSLL-KY-2022-001-01-01). The participants provided their written informed consent to participate in this study.

## Author contributions

All authors contributed to this trial concept and design. JF and XS designed the trial protocol. XW and MT wrote the first draft of the manuscript. MX, ZC, SP, HH, and YG critically revised the article, NC, JY, JJ, SQ, and SX took part in the recruiting process. All authors read and approved the final manuscript.

## Funding

The trial was supported by the Zhejiang Provincial TCM Science and Technology Program–Zhejiang Provincial TCM Modernization Special Project (2022ZX010).

## Conflict of interest

The authors declare that the research was conducted in the absence of any commercial or financial relationships that could be construed as a potential conflict of interest.

## Publisher’s note

All claims expressed in this article are solely those of the authors and do not necessarily represent those of their affiliated organizations, or those of the publisher, the editors and the reviewers. Any product that may be evaluated in this article, or claim that may be made by its manufacturer, is not guaranteed or endorsed by the publisher.

## Abbreviations

MDD: Major depressive disorder
IA: Intradermal acupuncture
AIA: Active intradermal acupuncture
SIA: Sham intradermal acupuncture
SSRIs: Selective serotonin reuptake inhibitors
RCT: Randomized controlled trial
MRI: Magnetic resonance imaging
rs-fMRI: Resting state-functional MRI
MRS: Magnetic resonance spectroscopy
HAMD-17: Hamilton depression rating scale-17
SDS: Self-Rating depression scale
PSQI: Pittsburgh sleep quality index
ACC: Anterior cingulate cortex
PFC: Prefrontal cortex.
